# Efficacy and Safety of Intravenous Ferric Carboxymaltose in Geriatric Inpatients at a German Tertiary University Teaching Hospital: A Retrospective Observational Cohort Study of Clinical Practice

**DOI:** 10.1155/2015/647930

**Published:** 2015-07-05

**Authors:** Matthias Bach, Tabea Geisel, Julia Martin, Bettina Schulze, Roland Schaefer, Garth Virgin, Juergen Stein

**Affiliations:** ^1^St. Elisabethen Krankenhaus, 60487 Frankfurt/Main, Germany; ^2^Interdisciplinary Crohn Colitis Centre Rhein-Main, 60594 Frankfurt/Main, Germany; ^3^Institute of Nutritional Science, University of Giessen, 35392 Giessen, Germany; ^4^Krankenhaus Sachsenhausen, Teaching Hospital of the J. W. Goethe University, 60594 Frankfurt/Main, Germany; ^5^Vifor Pharma Deutschland GmbH, 81379 Munich, Germany; ^6^Gastroenterology and Clinical Nutrition, Krankenhaus Sachsenhausen, Teaching Hospital of the J. W. Goethe University, 60594 Frankfurt/Main, Germany

## Abstract

Current iron supplementation practice in geriatric patients is erratic and lacks evidence-based recommendations. Despite potential benefits in this population, intravenous iron supplementation is often withheld due to concerns regarding pharmacy expense, perceived safety issues, and doubts regarding efficacy in elderly patients. This retrospective, observational cohort study aimed to evaluate the safety and efficacy of intravenous ferric carboxymaltose (FCM, Ferinject) in patients aged >75 years with iron deficiency anaemia (IDA). Within a twelve-month data extraction period, the charts of 405 hospitalised patients aged 65–101 years were retrospectively analysed for IDA, defined according to WHO criteria for anaemia (haemoglobin: <13.0 g/dL (m)/<12.0 g/dL (f)) in conjunction with transferrin saturation <20%. Of 128 IDA patients screened, 51 (39.8%) received intravenous iron. 38 patient charts were analysed. Mean cumulative dose of intravenous FCM was 784.4 ± 271.7 mg iron (1–3 infusions). 18 patients (47%) fulfilled treatment response criteria (≥1.0 g/dL increase in haemoglobin between baseline and hospital discharge). AEs were mild/moderate, most commonly transient increases of liver enzymes (*n* = 5/13.2%). AE incidence was comparable with that observed in patients <75 years. No serious AEs were observed. Ferric carboxymaltose was well tolerated and effective for correction of Hb levels and iron stores in this cohort of IDA patients aged over 75 years.

## 1. Introduction

Iron deficiency is one of the most common nutritional deficiencies in the elderly population as a whole, with a prevalence of approximately 10–15% in persons aged 65 and older and 35% in those aged 85 and above [1, 2]. Anaemia is defined according to World Health Organization (WHO) criteria as a haemoglobin level of <12 g/dL in women and <13 g/dL in men. While some 40–50% of hospitalised patients are anaemic, rates of up to 47% have also been found in patients living in nursing homes [1, 3, 4].

The aetiology of anaemia in the elderly is multifaceted. Major causes are reduced iron absorption as a result of drug interactions, diseases of the digestive tract, and/or chronic inflammation. Depletion of iron stores may also occur as a consequence of (mostly chronic) bleeding [5, 6]. However, anaemia is usually a result of iron deficiency, either absolute or functional (i.e., insufficient supply of iron to the erythroid marrow despite adequate iron stores) [7]. Geisel et al. reported 66% prevalence of anaemia in geriatric inpatients. 65% of cases were associated with iron deficiency (ID), mostly due to chronic infection, or ID combined with anaemia of inflammation (IDA/AI, 21.4%) [3]. While comorbidities and polypharmacy may be contributory factors, nutrition played only a limited role [2].

In the past, reduced haemoglobin levels were widely regarded as a normal consequence of the aging process [8]. However, data clearly show anaemia in the elderly population to be associated with a significantly higher risk of morbidity and mortality, even in the absence of concomitant illness [9, 10]. The impact of anaemia (even mild anaemia) on quality of life can be considerable in terms of energy levels, functional and cognitive capacity, and mobility [11, 12]. Typical anaemia symptoms such as physical weakness, tiredness, or dizziness may increase the risk of falls [13, 14], thus leading to increased hospitalisation and/or mortality [15, 16].

Even latent iron deficiency has been shown to significantly impair cognitive function and physical coordination [11, 17]. Motor deficiencies in older women increase drastically even when haemoglobin levels are in the lower normal range [18], increasing the risk of falls and resultant fractures [19].

In a recent review, Goodnough and Schrier recommend routine initial assessment of iron status in all elderly patients [20] and, once IDA is clearly ascertained, the commencement of oral iron supplementation as a therapeutic trial, aiming to correct anaemia and replenish iron stores. Under oral iron therapy, Hb levels can be expected to rise by a maximum of 1-2 g/dL every two weeks [21]. Replenishment of iron stores requires further supplementation for at least six months after anaemia correction. However, in elderly patients, therapy may be required for a longer period, since functional IDA (anaemia of inflammation, AI) leads to diminished intestinal iron absorption and slower bone marrow response [22, 23]. Adherence is often poor, particularly when patients have to consume numerous medications on a daily basis due to concomitant multimorbidity. Moreover, in geriatric patients, oral iron supplementation is often poorly tolerated, particularly as a result of abdominal discomfort, and poorly absorbed when malabsorptive conditions are present (see above).

Intravenous (i.v.) iron replacement therefore has considerable potential advantages for the treatment of elderly patients with IDA [21, 24]. Most i.v. iron formulations are effective, well tolerated, and associated with a lower incidence of serious adverse reactions (e.g., anaphylaxis) than most clinicians perceive [25–27].

While safety data for i.v. iron supplementation in geriatric patients are scarce, a recent retrospective study by Dossabhoy demonstrated iron dextran to be relatively safe and effective in elderly patients (mean, 70 years) with chronic kidney disease [28].

Ferric carboxymaltose (FCM, Ferinject, Vifor (International) Inc., St. Gallen, Switzerland) is a next-generation, parenteral, dextran-free, strong, and robust iron complex suitable for i.v. administration in individual doses up to 1000 mg and has been demonstrated to be safe and highly effective in the treatment of both mild and severe IDA in a large cohort of patients up to 70 years of age [29]. Apart from two recently published heterogenous chart analyses [28, 30], however, no data exist for patients over 70 years of age. Here, we report results of a monocentric retrospective chart data analysis designed to assess the safety and efficacy of i.v. iron as FCM in a cohort of patients aged over 75 years.

## 2. Study Design

This report presents a retrospective chart data analysis of patients admitted between March 2010 and March 2011 to the Geriatric Clinic of the St. Elisabethen Krankenhaus in Frankfurt, Germany, whose medical records were available. Aim of the analysis was to investigate the efficacy and safety of ferric carboxymaltose (Ferinject, Vifor Pharma). Data for analysis were extracted from the charts of elderly persons who had been admitted to the rehabilitation unit and treated according to the medical needs of their underlying disease in accordance with therapy requirements published in the German Physician's Circular (GPC). Patient charts included in the analysis were those of patients over 75 years of age with iron deficiency anaemia (IDA) whose hospital stay lasted at least 2 weeks. The 2-week minimum stay was chosen in order to allow time for optimal Hb correction even when i.v. iron was not administered until several days after admission.

Patients with Hb levels below 12 g/dL (women)/13 g/dL (men) and a TSAT value <20% were considered to have anemia associated with iron deficiency. Three subcategories were defined [31, 32]:

- Anaemia related to absolute iron deficiency (iron deficiency anaemia, IDA) was characterised by a decreased serum ferritin level (<30 *μ*g/mL) in combination with low serum CRP (<0.5 mg/dL).
- Anaemia caused by inflammation (AI) was defined by high ferritin levels (>100 *μ*g/mL) and increased CRP (≥0.5 mg/dL).
- Patients with ferritin levels between 30 *μ*g/mL and 100 *μ*g/mL and high CRP levels (≥0.5 mg/dL) were classified as having mixed anaemia (IDA/AI).

Patients were subdivided into two age groups, 76–85 years or >85 years, and additionally stratified by baseline ferritin values of ≤300 or >300 ng/mL. FCM (500 mg or 1000 mg) was diluted in normal saline and administrated i.v. over a 15–25 min period.

Data sets were excluded from analysis if any of the following factors were present: Hb < 8 g/dL, TSAT ≥ 20%, or serum albumin < 2.5 g/dL; known hypersensitivity to iron polysaccharide complexes or FCM; vitamin B_12_ or folic acid deficiency; anaemia due to causes other than iron deficiency; iron overload disorders (e.g., haemochromatosis); significant cardiovascular disease (including myocardial infarction during the six months prior to study inclusion); uncontrolled endocrinological or metabolic disorders; active infection, malignancy, or active liver disease.

Primary objective was to assess the safety of i.v. iron supplementation as FCM in hospitalised geriatric patients with anaemia. Secondary objective was to evaluate the efficacy of FCM in correcting iron deficiency and increasing Hb levels in this patient population.

### 2.1. Assessments

Safety and tolerability were assessed by analysing the incidence and severity of adverse events (AEs) at first dose of FCM and their relationship with FCM administration as recorded by the treating physician. Routine clinical laboratory safety parameters, vital signs, and physical parameters were also monitored and documented.

No primary efficacy endpoint was defined. Efficacy was assessed by changes in Hb levels as recorded after dose in patient charts during routine follow-up in the course of inpatient hospital care, and treatment responders were defined by an Hb increase of ≥1.0 g/dL from baseline until discharge from hospital. Laboratory parameters were determined at the local laboratory during the course of hospital care.

### 2.2. Statistical Analyses

Descriptive statistics were attained by calculation of arithmetical means, standard deviations, medians, and minimum and maximum values of all data. To test significance of all categorical variables, the chi^2^-test (Pearson) was performed. Arithmetical means were calculated with *t*-tests for dependent and independent samples, and correlations were determined using the Spearman Rho method. All outcomes with a minimum of *p* < 0.05 were considered significant. Missing values were disregarded in all statistical tests. All statistical analyses were performed using IBM SPSS Statistics 20SPSS statistical software.

## 3. Results

Of all subjects whose Hb values were recorded at admission (*n* = 386), 256 (66.3%; men 74.8%, women 62.9%) were anaemic. There was no correlation between age and Hb level. Of these, 154 (60.2%) were defined as having iron deficiency anaemia. Absolute IDA was found in only 7 (4.6%), while the majority were diagnosed with AI (128, 83.1%) or a combination of IDA and AI (19, 12.3%) indicated by high CRP and ferritin levels. Of those with IDA, only 51 (33.1%) received i.v. iron supplementation. After screening of patient records according to the criteria listed above, 38 charts were included in the retrospective analysis (subject characteristics shown in Table 1). Main reasons for noninclusion were a hospital stay shorter than 14 days and/or age below 76 years.

Mean cumulative dose of intravenous FCM was 784.4 ± 271.7 mg iron, administered in one to three infusions. In clinical practice, baseline requirements for replenishment of iron stores are commonly calculated using the Ganzoni formula [33]: total iron deficit = body weight [kg] × (target Hb − actual Hb) [g/L] × 0.24 + iron stores [mg]. Required dosage according to Ganzoni was 478.2–1724.0 mg iron (mean 1105.6 ± 299.2). Patients were, in fact, significantly undertreated, mean actual iron substitution being approximately 30% below the Ganzoni calculation.  Table 2 shows baseline iron parameters of subjects. Details of iron supplementation and Hb response are described in Table 3.

### 3.1. Safety

Intravenous iron as FCM was well tolerated, without clinically significant AEs. The most common AEs were transient increases of liver enzymes (APT and/or ALT) of greater than twice the upper normal level (*n* = 5, 13.2%), which completely resolved within a few days. Transient increases of liver enzymes are scarce but have also been reported in the FERGIcor [34] and PROCEED [35] trials. Skin and subcutaneous tissue disorders, such as rash, hand and/or feet oedema, and pruritus, which were considered study drug-related were reported in 3 patients (7.8%). Leukocyte counts and CRP levels were stable until hospital discharge in both age groups. There was no report of anaphylaxis, anaphylactoid reaction, or death or of any other SAE, during the twelve-month data extraction period.

### 3.2. Efficacy

The number of responders, defined by increase in Hb of ≥1.0 g/dL from baseline, was 18 out of 38 (47%). Mean Hb increased from 9.1 ± 1.30 g/dL (95% CI = 8.86; 9.26) at baseline to 9.5 ± 1.34 g/dL (95% CI = 9.25; 9.72) at 2 weeks after initial dose of FCM and continued to increase throughout the observation period, reaching 10.3 ± 1.63 g/dL (95% CI = 10.06; 10.58) at hospital discharge.

**Figure 1:**
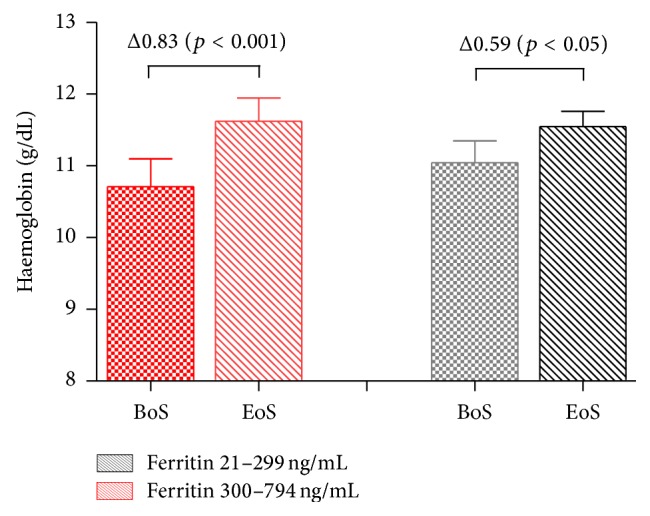


There was no significant difference between the two age groups (age 76–85 versus > 85 years) regarding percentage of responders or change in mean Hb levels (Table 3). There was also no significant difference between the two ferritin groups, defined as ≤300 versus >301 ng/mL, as regards magnitude of Hb response to i.v. iron (Figure 1).

## 4. Discussion

Studies show that morbidity and mortality can be reduced by treating ID in a wide range of conditions, including chronic heart failure [36, 37], chronic kidney disease [38, 39], cancer therapy [40, 41], inflammatory bowel disease [34, 42], and rheumatoid arthritis [38, 39]. Preoperative iron administration results in faster normalisation of Hb levels and lower incidences of postoperative blood transfusion, postoperative morbidities, and infection [19, 43–45].

IDA is common in geriatric patients, and even mild anaemia is associated with poorer outcomes, extended hospitalisation, and increased mortality. Geriatric patients should therefore be routinely assessed for anaemia and causes individually identified to allow prompt initiation of therapy. However, specific guidelines for the management of anaemia in the elderly are lacking.

While particularly in outpatients oral iron preparations are commonly preferred, intravenous ferric carboxymaltose (FCM, Ferinject) provides a new, convenient option for rapid iron substitution, especially in cases where oral substitution is ineffective or not tolerated. FCM allows single-dose i.v. application of 1000 mg iron in only 15 minutes. A recent meta-analysis of published clinical studies concluded that i.v. administration of FCM is safe and effective in the various populations studied [29]. Similar data have also been published for other iron formulations such as LMW ID [46], ferumoxytol [47], and isomaltose 1000 (only available in Europe) [35].

Our retrospective chart data analysis aimed to assess the safety of intravenous FCM in anaemic geriatric patients aged 75 years and over and to evaluate the efficacy of FCM in correcting iron deficiency and Hb levels in this patient population.

Based on the Ganzoni formula, subjects were undertreated, receiving only two-thirds of the Ganzoni-calculated dose for iron store replenishment. This comes as no surprise. The complexity of the Ganzoni formula makes it impractical for everyday use. Consequently, iron requirements are often freely estimated, with physicians instinctively erring on the side of caution (or nonuse of i.v. iron) due to conceived risks of i.v. iron, especially in elderly patients. Reduced haemoglobin levels are still widely (erroneously) assumed to be an acceptable consequence of aging (see above); therefore target Hb levels are frequently lower in geriatric than in nongeriatric patients. A novel and simple dosing method based on body weight in kg and baseline Hb was recently shown by Evstatiev et al. [34] to be safe and effective in patients with inflammatory bowel disease. The calculation table used in this study (Table 4) can be used as a practical dosage guide.

Despite being undertreated, 47% of subjects were treatment responders, achieving a clinically relevant increase in Hb level of ≥1.0 g/dL after receiving iron as FCM. Clinically relevant increases (from 10.74 g/dL at baseline to 11.68 g/dL at discharge) were observed in the majority of patients, attributable to the administration of i.v. iron as FCM. These findings compare favourably with data published recently by Dossabhoy et al. [28].

The current study also examines the effectiveness of i.v. iron in the context of “*an increase in ferritin level accompanied by a decrease in TSAT that is suggested to lead to inflammation-mediated reticuloendothelial blockade*” [38]. Data from the 2007 Drive Study, a well-designed, open-label, randomised, controlled, multicentre trial in dialysis patients, clearly demonstrated that ferritin values did not predict responsiveness to intravenous iron and that i.v. iron supplementation (here, ferric gluconate at doses of up to 200 mg) was successful even at ferritin levels of 500–1200 ng/mL [38]. These findings were echoed by our results. Other studies have also shown ferritin to be unpredictive of iron responsiveness when ferritin is <500 ng/mL. Therefore, ferritin cannot be recommended as a guiding parameter for therapy except when being low (<300 ng/mL), in which case it is highly predictive of iron deficiency [48, 49].

Our study has three main limitations. Firstly, due to its retrospective design, only laboratory parameters collected as clinical routine were assessable. Due to prohibitive costs, concentrations of hepcidin, a liver-made peptide which is thought to regulate iron metabolism, were not routinely measured; thus, any correlation between hepcidin concentration, degree of functional iron deficiency, and response to i.v. iron could not be addressed. Secondly, after exclusion of patient data with confounding conditions and after screening for advanced age, IDA, and a hospital stay of at least two weeks, the number of data sets analysed, at 38, was relatively small. Thirdly, the study focused on hospitalised geriatric patients rather than outpatients. Results may therefore lack consistency and may be unrepresentative for elderly patients as a whole. The results should therefore be interpreted with caution.

## 5. Conclusions

The importance of IDA in the general population is increasingly recognised. Guidelines for the detection, evaluation, and management of IDA recommend i.v. supplementation as the preferred route in a variety of medical and surgical situations. However, safety and efficacy data for i.v. iron in elderly and very elderly populations have been lacking. The data presented here suggest that i.v. iron supplementation, even in cumulative doses of up to 1500 mg in one to three infusions, is safe and effective in this population.

## Figures and Tables

**Table 1:** Patient characteristics: main reason for hospitalisation and length of stay.

	All, *n* = 38	76–85 yrs, *n* = 17 (%)	>85 yrs, *n* = 21 (%)
Age (years)	85.9 ± 5.05	81.24 ± 2.94	89.71 ± 2.66
Median (range)	86.5 (76–96)	82.0 (76–85)	90.0 (86–96)
Fractures	18	7	11
Cardiovascular disease	6	4	2
Disturbances of gait and mobility	5	3	2
Digestive tract diseases	2	1	1
Neoplasm	1	0	1
Infectious diseases	3	0	3
Cerebrovascular disease	3	2	1
Hospital stay (d)			
mean ± SD	27.2 ± 8.9	26.9 ± 8.9	27.2 ± 8.9
(median; min–max)	(26; 14–53)	(27; 14–53)	(26; 14–50)

**Table 2:** Baseline iron parameters of all patients aged >75 years, subdivided into age groups.

Age group	Number of patients	Hb (g/dL)	Ferritin (*µ*g/L)	TSAT (%)	CRP (mg/dL)
		mean ± SD	mean ± SD	mean ± SD	mean ± SD
		(median; min–max)	(median; min–max)	(median; min–max)	(median; min–max)
76–85 years	17	10.42 ± 0.85	194.52 ± 144.49	9.59 ± 3.71	7.16 ± 10.33
		(10.3; 8.8–11.8)	(160.0; 21–714)	(10.00; 4–17)	(4.1; 0.2–49.0)

>85 years	21	11.00 ± 1.52	255.42 ± 208.64	9.6 ± 2.78	5.49 ± 7.03
		(10.7; 8.8–13.5)	(163.0; 29–796)	(10.00; 5–10)	(3.6; 0.2–28.7)

All	38	10.74 ± 1.30	228.2 ± 185.24	9.61 ± 3.23	6.41 ± 9.04
		(10.5; 8.8–13.5)	(161.50; 21–796)	(9.6; 4–17)	(3.9; 0.2–49.0)

**Table 3:** Amount of iron supplementation and Hb response.

Age group	Calculated iron requirement (mg) mean ± SD (median; min–max)	Supplemented iron (mg) mean ± SD (median; min–max)	Number of infusions	Hb BoS (g/dL) mean ± SD (median; min–max)	Hb EoS (g/dL) mean ± SD (median; min–max)	ΔHb (g/dL) mean ± SD (median; min–max)	% responder (ΔHb >1 g/dL)
76–85 years	1210.4 ± 308.4 (1205.6; 1205.6–1724.0)	763.2 ± 297.7 (500; 500–1500)	(1–3)	10.42 ± 0.85 (10.3; 8.8–11.8)	11.31 ± 1.17 (11.9; 9.5–14.3)	0.54 ± 1.49 (0.9; 0.0–3.2)	8/17 (41%)

>85 years	1022.5 ± 263.7 (1046.4; 500.0–1493.6)	770.8 ± 249.1 (500; 500–1000)	(1-2)	11.00 ± 1.52 (10.7; 8.8–13.5)	11.75 ± 0.86 (11.9; 10.2–13)	0.69 ± 1.10 (0.9; 0.0–2.6)	10/21 (48%)

All	1105.6 ± 299.2 (1085.6; 478.2–1724.0)	784.4 ± 271.7 (500; 500–1500)	(1–3)	10.74 ± 1.30 (10.5; 8.8–13.5)	11.68 (11.35; 8.8–14.3)	0.62 ± 1.58 (0.9; 0.0–3.2)	18/38 (47%)

BoS: beginning of study; EoS: end of study.

**Table 4:** Novel iron dosing strategy based on body weight and Hb levels as proposed by Evstatiev et al. [34].

Haemoglobin g/L	Body weight <70 kg	Body weight ≥70 kg
≥100	1000 mg	1500 mg
70–100	1500 mg	2000 mg
<70	2000 mg	2500 mg
